# Neurological recovery across a 2-cm gap of radial nerve repair using end to end suture following supracondylar humerus fracture: Case report

**DOI:** 10.1016/j.ijscr.2021.105896

**Published:** 2021-04-16

**Authors:** Muh. Ihsan Kitta, Mirza Ariandi, Yosua Adi Nugroho, Adhika Nur, Ferdinand Arden

**Affiliations:** a Department of Orthopaedics and Traumatology, Faculty of Medicine of Hasanuddin University, Makassar, Indonesia; b Lecturer of Medical Faculty of Muhammadiyah University, Makassar, Indonesia

**Keywords:** Case report, Supracondylar fracture, Radial nerve, Transection

## Abstract

**Introduction and Importance:**

Traumatic laceration of the radial nerve following supracondylar humerus fracture in the pediatric population is highly uncommon. Transection of the radial nerve is rare, and approximately 12.7% of all nerve injuries are categorized as neurapraxia that will resolve spontaneously.

**Case presentation:**

We report a rare case of a 9-year-old boy presented with a right wrist drop after fell on an outstretched hand two weeks before. The radiological finding revealed a posteriorly displaced supracondylar humerus fracture. Intraoperatively, we found the radial nerve had lost its continuity and interposed by fibrotic tissue. We resect and do a direct repair of the nerve along with fixation of the fracture. At 4-months postoperative follow-up, the patient could fully extend his right thumb and wrist but retain a tingling sensation in the radial nerve distribution. At 1-year follow up the motor and sensory functions were normal without any complication.

**Clinical Discussion:**

Transection of radial nerve following the supracondylar fracture represents a rare case. The radial nerve was transected and interposed by scar tissue at the fracture site level, preventing spontaneous nerve regeneration. In a case of nerve transection, surgical repair is indicated.

**Conclusion:**

In the case of supracondylar humerus fracture with posterior displacement, radial nerve transection could be associated with injuries. Authors favour nerve exploration with fracture stabilization performed as soon as possible when nerve injury is suspected. Appropriately managed, the outcome in the pediatric population is highly favourable.

## Highlights

• Complete laceration of the radial nerve following supracondylar humerus fracture is a rare finding.
• Radial nerve injury with wrist-drop is seen two weeks after the injury.
• The sensory disturbance is shown as anaesthesia in the radial nerve distribution.
• Neurorrhapy was done in conjunction with the open reduction internal fixation of the fracture.

## 1 Introduction

Traumatic laceration of the radial nerve following supracondylar humerus fracture (SCF) in the pediatric population is a rare case. The neurologic deficit in SCF is reported in about 10%–20% of cases, and up to 49% of nerve injury is reported in Gartland Type III Fractures [1]. Nerve injuries associated with SCF are mostly classified as neurapraxia, and up to 80% could recover spontaneously [1,2].

## 2 Case presentation

**Fig. 1:**
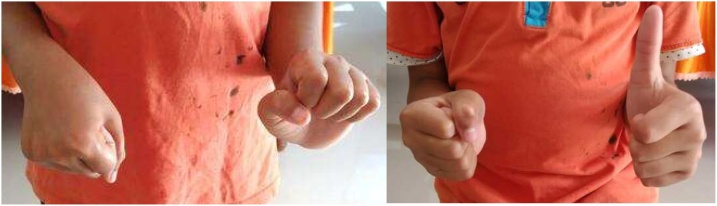
Preoperative clinical finding showing right wrist-drop: inability to extend wrist and thumb.

**Fig. 2:**
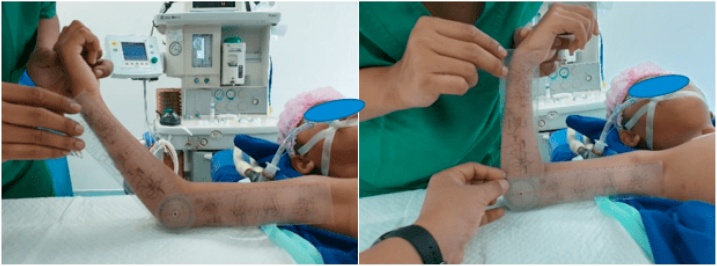
Preoperative clinical finding showing elbow’s range of motion was limited (60° to 90°).

**Fig. 3:**
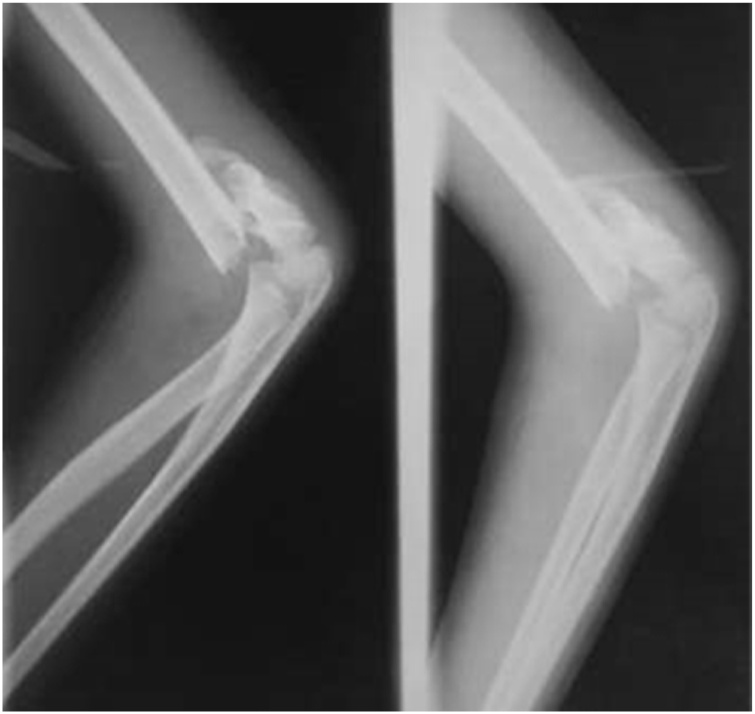
Radiographs were showing supracondylar humerus fracture (Gartland type III).

A right-handed 9-year-old boy fell on an outstretched hand two weeks before admitted to the hospital. The patient presented with a right wrist-drop and right elbow flexion stiffness. On the physical examination, the patient cannot extend his right wrist and thumb. There was also sensory deficit, anaesthesia sensation over the radial nerve distribution and hypoesthesia sensation on the ulnar nerve distribution. The median and ulnar nerve examinations were standard. The distal pulsation is solid and regular. The right elbow joint was stiff with a limited flexion-extension range of motion (60–90°). Radiographs showed a displaced SCF classified as Gartland type III. The parents first sought treatment from a traditional bonesetter before came to the hospital (Fig. 1, Fig. 2, Fig. 3).

**Fig. 4:**
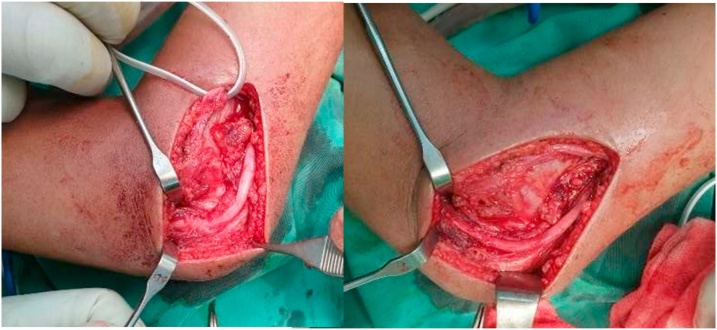
Ulnar nerve exploration via medial approach: the ulnar nerve is intact but entrapped in fibrous tissue.

**Fig. 5:**
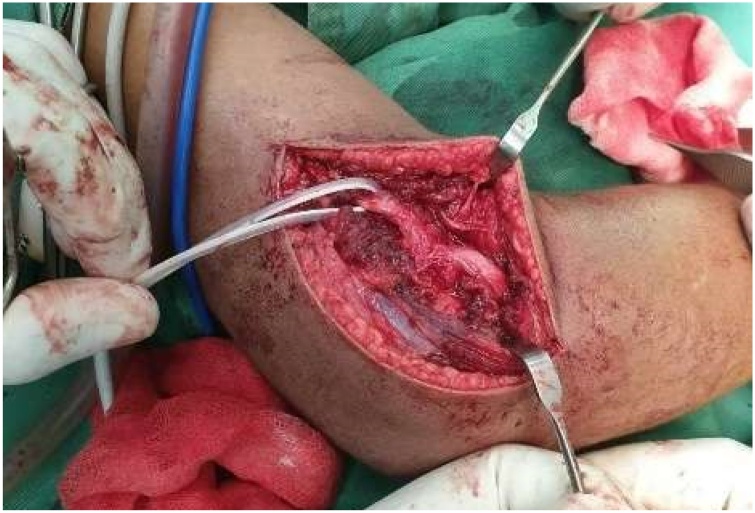
Radial nerve exploration via lateral approach: the radial nerve has lost its continuity and interposed by fibrotic tissue.

**Fig. 6:**
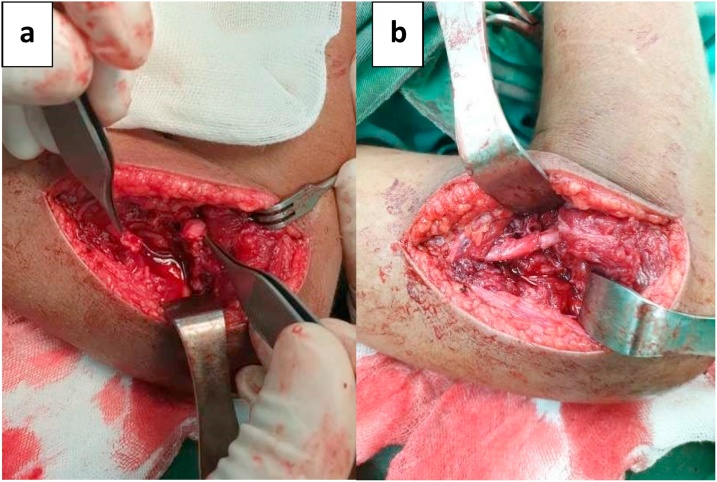
Preparation of direct radial nerve repair. a. Resection of scarred nerve and fibrotic tissue, 2 cm gap in nerve observed. b. Direct repair of the defect with end-to-end neurorrhaphy without tension.

**Fig. 7:**
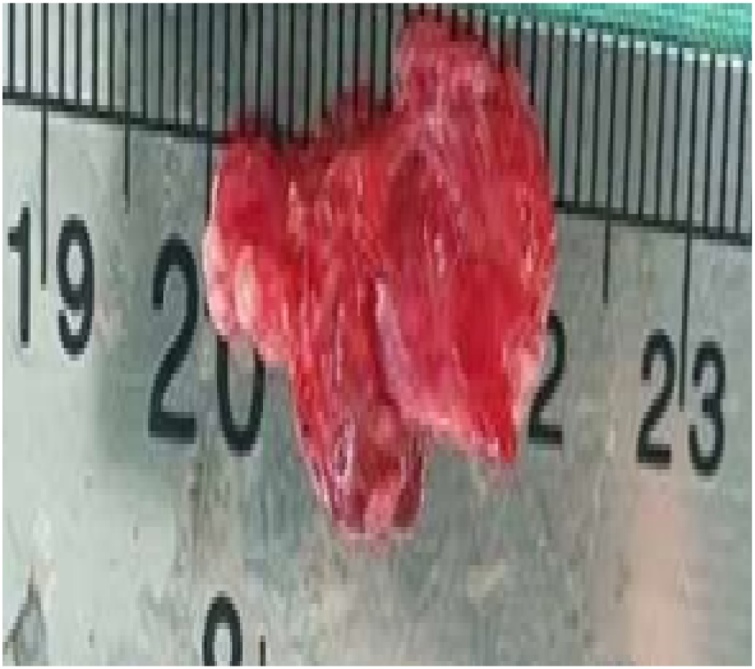
2 cm of radial nerve resection.

Surgical management is preferred for this case, and we decided to explore the nerve while simultaneously reduce and stabilize the fracture. First, we explored the ulnar nerve with a medial approach and found the ulnar nerve was entrapped in fibrous tissue and carefully released it. Then the lateral approach is utilized to explore the radial nerve. We found the radial nerve has lost its continuity and interposed by fibrotic tissue. The scar and fibrous tissue were carefully resected, leaving a 2 cm gap in the nerve. We approximate the nerve with end-to-end neurorrhaphy after the fracture was reduced and stabilized with 3-Kirschner wire. Postoperatively, the elbow was kept in long-arm cast with elbow flexed 90° to avoid nerve stretching for six weeks. The distal vascular status is good after the operation and cast application (Fig. 4, Fig. 5, Fig. 6, Fig. 7).

**Fig. 8:**
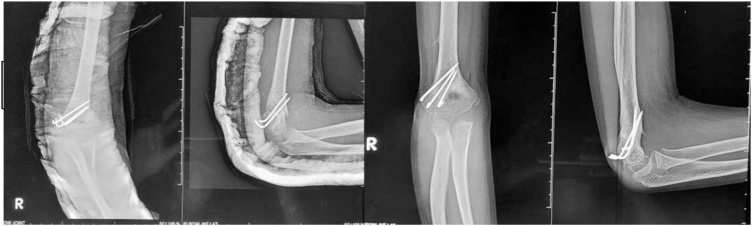
a. Postoperative radiographs of supracondylar fracture of humerus showing a reduced fracture with three lateral K-wire fixations; b. four months of postoperative radiographs were showing complete fracture union.

**Fig. 9:**
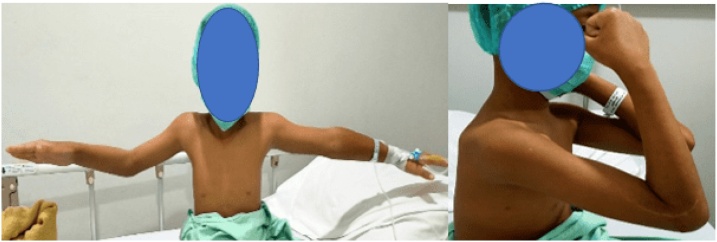
Postoperative clinical finding at four months showing elbow’s range of motion was improved (20°–115°).

**Fig. 10:**
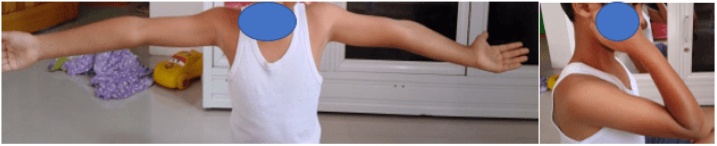
Postoperative clinical finding at ten months showing improved elbow range of motion.

At the 4-month follow-up, the fracture was united. The elbow range of motion has improved; 20° to 115° elbow flexion is seen. The neurological status showed an improvement. The patient could extend his right thumb and wrist but developed a tingling sensation in the posterior forearm, extending to the dorsum of the hand and lateral three and a half fingers; the tingling was triggered by full extension of the elbow. Concerning the radial nerve condition, we did not release and manipulate the elbow. At 1-year follow-up, the motor and sensory function fully recovered (Fig. 8, Fig. 9, Fig. 10).

## 3 Discussion

The complete radial nerve laceration associated with SCF is an uncommon case. The frequency of neurological deficit in children is about 10%–20%, and as high as 49% in Gartland Type III fracture [1].

Neuropraxia is the mildest type of nerve injury defined by a temporary blockage of nerve conduction, commonly found after traction or compression injuries [1,2]. Some surgeons recommend conservative management because 80% of neuropraxia often resolves spontaneously [3,4].

The nerve can be injured on the accident, during fracture manipulation, during surgery, or through entrapment by callus formation [5,6]. The radiographs showed a fracture of the SCF classified as Gartland type III with distal fragment completely displaced to the posterior side. The median nerve and its anterior interosseous nerve branch are at risk and commonly involved in the posterolateral fracture displacement. In contrast, the radial nerve is commonly involved with posteromedial displacement, which is reported at a rate of 2–3 times that of posterolateral displacement [1,4].

Surgical intervention may be indicated in nerve transection cases, fracture displacement associated with nerve injury, or failed conservative treatment in 3–6 months [3,5,6,7]. In our patient, surgical treatment was required because of the fracture's severe displacement, which could damage the nerve. Early surgical treatment and repair of an injured nerve can be crucial when neurotmesis is present [5].

Complete transection of the radial nerve is a rare case that only has been reported four times in the literature [8]. The nerve can be repaired by direct suturing or nerve grafting [5]. In nerve transection, the direct end-to-end repair is the method of choice. In cases where direct repair of the peripheral nerve is complicated due to segmental defects or ‘gaps’ in the nerve, nerve grafting can be one option [5].

The author decided to explore the nerve while simultaneously reduce and stabilize the fracture. Intraoperatively, the radial nerve was found to be transected and interposed by scar tissue at the level of the fracture site. The radial nerve was released and resected about 2 cm. The longest defect of radial nerve that ever published was 18 cm [8]. The Zachary studies reported that from 113 cases of repair by direct suture, the resected nerve's maximum length is 5 cm [9].

The nerve can be repaired by direct suturing or nerve grafting, depending on the gap length [9]. We recommend the end-to-end neurorrhaphy without tension as the method of choice. Tension at the repair site could result in ischemia, connective tissue proliferation, and scar formation that impair or prevent the regenerating axons [10]. A summary of the available literature on nerve injuries associated with pediatric extension SCF demonstrates that nerve injuries should recover within six months from their initial injury [11].

In this case, At the 4-month follow-up, the motor status was fully recovered. The patient could extend his right thumb and wrist but developed a tingling sensation in the posterior forearm and dorsal side of the hand, especially in three and a half lateral digits. Concerned about the radial nerve condition, the author chose not to do manipulation of the remaining contracture. At 1-year follow-up, the motor and sensory function fully recovered.

In some literature, Amillo and Mora reported 6 cases of radial nerve injury in children with elbow fractures; all radial nerves were found to be in continuity and were treated by neurolysis. Functional result obtained in those operated upon within 12 months [12]. Martin et al. documented a case of radial nerve laceration with extreme retraction after a closed SCF of the elbow and treated with nerve grafting. One year after the operation, the patient demonstrated a total return of radial nerve function [8]. Finally, Bertelli et al. describe seven children with radial nerve injury after SCF. An average recovery time of 19.9 months after surgery, with entire wrist, thumb, and finger extension, was documented [11].

## 4 Conclusion

The radial nerve involvement in the pediatric SCF is rare, with neuropraxia as the most common finding. However, in our case, we found intraoperatively that the radial nerve is completely transected thus decided to do primary repair. We recommend a surgical fixation of the bone and primary nerve repair using non-tension suturing in this kind of case

## Declaration of Competing Interest

There is no conflict of Interest on all Authors.

## Sources of funding

The author(s) received no financial support for the research, authorship, and/or publication of this article.

## Ethical approval

The study is exempt from ethnical approval from our institution.

## Consent

Written informed consent was obtained from the patient for publication of this case report and accompanying images. A copy of the written consent is available for review by the Editor-in-Chief of this journal on request.

## Author contribution

**Muhammad Ihsan Kitta:** Conceptualization, Methodology, Software, Supervision, Data Curation.

**Mirza Ariandi** : Data curation, Writing - Original draft preparation, Supervision

Yosua Adi Nugroho: Visualization, Investigation, Writing, Funding acquisition.

**Adhika Nur Syamsul**: Visualization, Investigation, Writing, Funding acquisition.

**Ferdinand Arden:** Writing, Funding acquisition.

## Registration of research studies

researchregistry6580 available at: https://www.researchregistry.com/browse-the-registry#home/.

## Guarantor

dr. Muhammad Ihsan Kitta, M. Kes, Sp. OT (K).

## Provenance and peer review

Not commissioned, externally peer-reviewed.

## Other relevant information

The case report complies with SCARE Guidelines [12].
